# Commentary: Exploring the association between immune-inflammation index and carotid plaque formation: a cross-sectional study in a large Chinese health screening population

**DOI:** 10.3389/fendo.2025.1763899

**Published:** 2026-01-12

**Authors:** Zhiwei Hu, Hua Dong, Xiaozhu Huang

**Affiliations:** 1 Department of Endocrinology, The First People’s Hospital of Jiashan, Jiashan Hospital Affiliated of Jiaxing University, Jiaxing, Zhejiang, China; 2 The First People’s Hospital of Jiashan, Jiashan Hospital Affiliated of Jiaxing University, Jiaxing, Zhejiang, China

**Keywords:** aggregate index of systemic inflammation, atherosclerosis, carotid plaque, systemic immune- inflammation index, systemic inflammation response index

## 1 Introduction

Sun et al. (1) conducted a large-scale cross-sectional study with 9,503 Chinese health screening participants. They investigated the associations between composite inflammatory indices—systemic immune-inflammation index (SII), systemic inflammation response index (SIRI), and aggregate index of systemic inflammation (AISI)—and carotid plaque formation. They identified AISI as the most robust inflammatory biomarker for carotid plaque prediction and developed a four-variable model—including age, fasting glucose, AISI, and diabetes mellitus—that showed favorable discriminative performance (AUC = 0.744 in the validation cohort). Notably, TC and LDL-C, both well-known causal risk factors for atherosclerosis, showed no significant association with carotid plaque in univariate analysis (P = 0.118 and P = 0.345, respectively). This finding contradicts both the well-validated pathophysiology of atherosclerotic cardiovascular disease (ASCVD) and recent high-impact evidence confirming LDL-C’s role in plaque development. Additionally, the study relied on single-timepoint measurements of inflammatory indices, which limits its ability to capture the dynamic nature of systemic inflammation. This dynamic process is critical in carotid plaque initiation and progression, as supported by longitudinal studies (3).

## 2 Discrepancy between TC/LDL-C and carotid plaque: challenging established ASCVD pathophysiology

The guidelines of ESC and ACC/AHA both agree that LDL-C is a major risk factor for atherosclerotic plaques during the entire process of plaque formation and development (2, 3). Separate meta-analyses of over 200 prospective cohort studies, Mendelian randomization studies, and randomized trials demonstrate a remarkably consistent dose-dependent log-linear association between the absolute magnitude of exposure of the vasculature to LDL-C and the risk of ASCVD (4). This effect appears to increase with longer duration of LDL-C exposure. Sun et al.’s failure to find an association between TC/LDL-C and carotid plaque suggests possible methodological or contextual limitations obscuring this relationship.

Three key factors may explain this discrepancy: (1) Unmeasured lipid subfractions: The study assessed only TC and LDL-C, omitting sdLDL-C and apolipoprotein B (apoB). Studies have shown that higher levels of sdLDL are significantly associated with an increased risk of ASCVD because sdLDL has low affinity for LDL receptors and a long residence time in the plasma (5). (2) Lack of adjustment for lipid-modifying interventions: The study did not report on statin use or adherence to lipid-lowering diets. These factors may weaken the observed lipid-plaque association. (3) Insensitive plaque detection criteria: Using the Mannheim Consensus criteria (focal thickening ≥1.5mm) may miss early subclinical plaques, where LDL-C-mediated lipid infiltration is most responsive to lipid levels. A review found that early plaque burden correlates strongly with lipid markers when detected by high-resolution imaging, highlighting the need for more sensitive detection methods (6).

To address this limitation, three improvements are recommended. First, incorporate lipid subfraction analysis by measuring sdLDL-C and apoB. Second, stratify by lipid-modifying interventions by adjusting analyses for statin use and the dietary inflammatory index. Third, enhance plaque characterization by combining structural ultrasound with functional assessments such as plaque attenuation values.

## 3 Single-timepoint inflammatory index measurement: limitations in capturing dynamic inflammatory burden

Systemic inflammation is a dynamic process. Transient elevations caused by acute, self-limited conditions differ fundamentally from chronic low-grade inflammation in their clinical relevance to atherosclerosis. A study (7) analyzed data from 3,927 individuals without carotid atherosclerosis who underwent yearly health check-ups at the Department of Health Management of Nanfang Hospital between 2011 and 2018. The median follow-up was 4.42 years. The study demonstrated that the continuous SIRI, a composite of neutrophils, monocytes, and lymphocytes, significantly predicted carotid plaque incidence. This predictive effect was more pronounced in subgroups without hypertension, diabetes, or hyperlipidemia. This supports the notion that persistent inflammatory burden—not isolated elevations—drives plaque progression and vulnerability.

The single-measure approach in Sun et al.’s study has two critical limitations. First, it may misclassify inflammatory status. A single elevated AISI or SIRI value could reflect acute inflammation, such as a recent upper respiratory tract infection not excluded by the 3-month criterion, rather than chronic pathology. Chronic inflammation of the vascular wall is a central pathogenesis of atherosclerosis (8). This may lead to an overestimation of the association between inflammation and prevalent plaque. Second, static baseline measurements lack predictive validity. They fail to capture inflammatory trajectories, such as progressive increases in AISI over time, which better predict incident plaque.

To address these limitations, we propose three improvements: (1) use a prospective longitudinal design with annual measurements of inflammatory indices to track changes and their links to new carotid plaques; (2) create composite scores that incorporate long-term inflammatory exposure, such as the average AISI over 2 years, to better capture chronic burden; (3) exclude participants with acute inflammation identified by questionnaires or biomarker panels (C-reactive protein>10 mg/L), isolating the effects of chronic inflammation.

## 4 Discussion

Sun et al.’s study provides valuable insights into AISI’s role in carotid plaque prediction. However, the LDL-C-carotid plaque discrepancy and the limitation of single-timepoint inflammatory measurements reduce its clinical translational value. Resolving the LDL-C paradox requires integrating lipid subfraction analysis (sdLDL-C, apoB) and adjusting for lipid-modifying treatments such as statins and PCSK9 inhibitors—steps that align findings with established ASCVD biology and enhance clinician trust in the four-variable model. Consequently, the model complements, rather than contradicts, evidence-based lipid-lowering strategies.

Additionally, longitudinal monitoring of inflammatory indices—consistent with Nai et al.’s study (6) that tracked SIRI and carotid plaque over 4.42-year follow-up—will strengthen the model’s ability to identify individuals at persistent plaque progression risk. Unlike single-timepoint measurements, tracking dynamic inflammatory changes and overall burden improves risk stratification. This also helps guide timely interventions to reduce plaque vulnerability.

Addressing these limitations through lipid subfraction analysis, cumulative inflammatory metrics, and prospective data collection will refine the study’s conclusions and better guide clinical practice, bridging the gap between inflammatory biomarker research and evidence-based ASCVD prevention to improve risk identification and management of carotid atherosclerosis.
